# Standardization and validation of a panel of cross-species microsatellites to individually identify the Asiatic wild dog (*Cuon alpinus*)

**DOI:** 10.7717/peerj.7453

**Published:** 2019-09-02

**Authors:** Shrushti Modi, Bilal Habib, Pallavi Ghaskadbi, Parag Nigam, Samrat Mondol

**Affiliations:** Wildlife Institute of India, Chandrabani, Dehradun, Uttarakhand, India

**Keywords:** Dhole, Individual identification, Non-invasive sampling, Population parameters, Maharashtra tiger landscape

## Abstract

**Background:**

The Asiatic wild dog or dhole (*Cuon alpinus*) is a highly elusive, monophyletic, forest dwelling, social canid distributed across south and Southeast Asia. Severe pressures from habitat loss, prey depletion, disease, human persecution and interspecific competition resulted in global population decline in dholes. Despite a declining population trend, detailed information on population size, ecology, demography and genetics is lacking. Generating reliable information at landscape level for dholes is challenging due to their secretive behaviour and monomorphic physical features. Recent advances in non-invasive DNA-based tools can be used to monitor populations and individuals across large landscapes. In this paper, we describe standardization and validation of faecal DNA-based methods for individual identification of dholes. We tested this method on 249 field-collected dhole faeces from five protected areas of the central Indian landscape in the state of Maharashtra, India.

**Results:**

We tested a total of 18 cross-species markers and developed a panel of 12 markers for unambiguous individual identification of dholes. This marker panel identified 101 unique individuals from faecal samples collected across our pilot field study area. These loci showed varied level of amplification success (57–88%), polymorphism (3–9 alleles), heterozygosity (0.23–0.63) and produced a cumulative misidentification rate or PID_(unbiased)_ and PID_(sibs)_ value of 4.7 × 10^−10^ and 1.5 × 10^−4^, respectively, indicating a high statistical power in individual discrimination from poor quality samples.

**Conclusion:**

Our results demonstrated that the selected panel of 12 microsatellite loci can conclusively identify dholes from poor quality, non-invasive biological samples and help in exploring various population parameters. This genetic approach would be useful in dhole population estimation across its range and will help in assessing population trends and other genetic parameters for this elusive, social carnivore.

## Introduction

The Asiatic wild dog or dhole (*Cuon alpinus*) is a highly elusive, endangered, social canid distributed in south and southeast Asia (Johnsingh, 1982; Durbin et al., 2004) occupying a range of habitat types including alpine, temperate, sub-tropical and tropical forests (Durbin et al., 2004). Driven by habitat loss, prey depletion, disease transmission from domestic dog, human persecution and interspecific competition (Hayward, Lyngdoh & Habib, 2014; Kamler et al., 2015), dholes are currently found in about 75% of their historical global range (Durbin et al., 2004; Kamler et al., 2015). Global dhole population is roughly estimated to be about 4,500–10,500 with only 949–2,215 mature individuals, but accurate estimates and population trends are not available from any part of its range (Kamler et al., 2015). They are considered as ‘Endangered’ by IUCN under criteria C2a(i) and included in Appendix II of the Convention on International Trade in Endangered Species. The Indian subcontinent currently retains majority of the remaining dhole populations (Kamler et al., 2015) in the Western Ghats and central Indian forests (Karanth et al., 2009), along with smaller populations in the Eastern Ghats (Karanth et al., 2009), northeast India (Gopi, Lyngdoh & Selvan, 2010; Lyngdoh et al., 2014) and Himalayan region (Bashir et al., 2014). The species has faced about 60% decline in their historical distribution in the subcontinent (Karanth et al., 2010).

Given the current anthropogenic disturbance scenario across its range, the future survival of this monotypic genus depends on integrated conservation measures involving detailed, accurate information on ecology, demography and genetics. However, generating reliable information for this elusive, forest-dwelling and pack-living canid at landscape scale is challenging. Traditional ecological techniques such as regular photographic capture approach are ineffective for dholes due to absence of unique coat patterns and their monomorphic forms, and physical tagging methods are impractical at landscape scales due to logistical difficulties, high costs and small numbers of captures possible. In this context, genetic tools have tremendous potential to generate critical information such as population size estimation (Mondol et al., 2009b), phylogeography (Luo et al., 2014; Waits et al., 1998), pack dynamics and reproductive fitness (Sillero-Zubiri, Gottelli & Macdonald, 1996; Girman et al., 1997), dispersal patterns (Epps et al., 2007; Gour et al., 2013) for elusive species conservation across large landscapes (Mondol, Bruford & Ramakrishnan, 2013). The ability to identify individuals from non-invasive samples collected over large space provides a feasible option to generate detailed information on elusive, forest-dwelling dholes as they cannot be identified using other approaches.

In this study, we addressed key methodological issues related to selection and standardisation of a set of molecular markers for individual identification of dholes. Subsequently, we tested these markers on field-collected dhole samples from five protected areas of the central Indian landscape in the state of Maharashtra, India for individual identification. In addition to the utilisation of these markers in dhole population estimation at landscape level, we believe that this approach has wider relevance in non-invasive, faecal DNA based population assessments of many other low density, elusive, wide-ranging species.

## Methods

### Research permits and ethical considerations

All required permissions for fieldwork and sampling were provided by the Maharashtra Forest Department (Permit No. 09/2016). The entire study was non-invasive through field-collected faecal samples, and thus did not require any ethical clearance from the institute. Reference dhole blood samples (*n* = 4) were collected as part of another ongoing study in Tadoba-Andhari Tiger Reserve (TATR; Permit no. SPP-12/2016), where blood sampling was conducted during radio collaring of dholes.

### Study area

The study was focused in five protected areas Melghat Tiger Reserve (MTR), Pench Tiger Reserve (PTR), Navegaon-Nagzira Tiger Reserve (NNTR), TATR and Umred-Karandhla Wildlife Sanctuary (UKWLS) of the central Indian landscape in the state of Maharashtra, India. The entire area is a complex of forested areas (core zone) with different levels of connectivity. NNTR and PTR are geographically closer as compared to MTR–NNTR and MTR–PTR. MTR and PTR are part of the Satpura-Maikal-Pench corridor in the Satpura-Maikal landscape. The forest type is of dry deciduous to moist deciduous nature with major vegetation consisting of *Tectona grandis, Anogeissus latifolia, Lagerstroemia parviflora, Terminalia* spp., *Heteropogon contortus, Themeda quadrivalvis, Cynodon dactylon* etc.

### Field sampling

**Figure 1:**
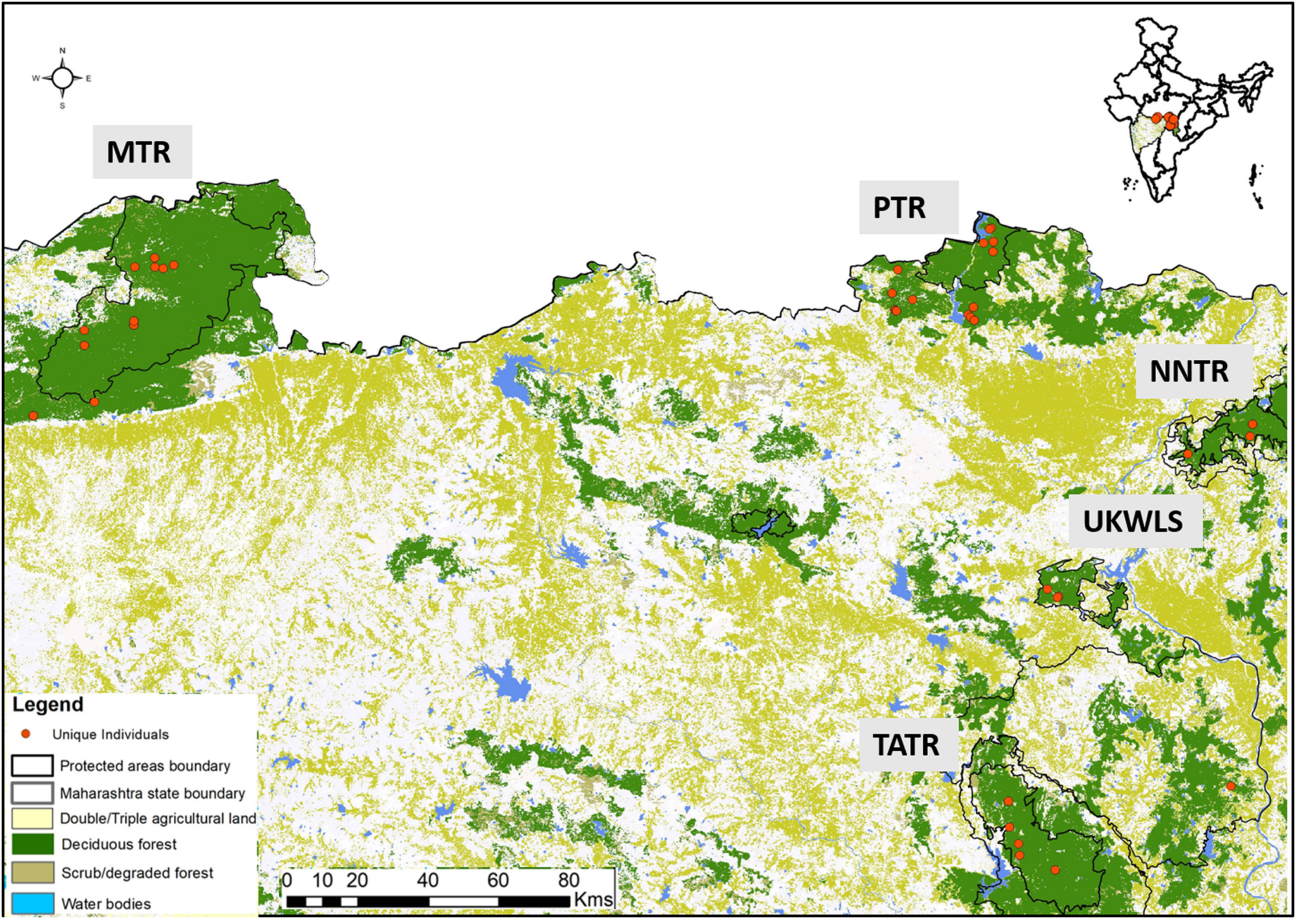
Protected area map with locations of unique genotypes identified in the study area of Maharashtra, India. The study area names are as following: MTR, Melghat Tiger Reserve; PTR, Pench Tiger Reserve; TATR, Tadoba-Andhari Tiger Reserve; NNTR, Navegaon-Nagzira Tiger Reserve; UKWLS, Umred-Karandhla Wildlife Sanctuary.

Dholes prefer dense forested habitats (Johnsingh, 1985) where the social groups defaecate in communal latrine sites mostly found on the junctions of roads/trails (Johnsingh, 1982). Their elusive nature and highly social behaviour present unique challenges in scat sampling for individual identification. In this study, sampling was conducted through intensive foot and vehicle surveys covering the entire study area. We sampled a total of 49 latrine sites covering five protected areas. Once a latrine site was found only fresh scats were targeted for collection. One bolus from each fresh scat was collected assuming it to derive from one individual. Separate gloves were used to collect each sample. All samples were collected directly in wax paper and stored in separate ziplock bags. Once brought to the field station, the sample containing ziplock bags were temporarily stored in a large box containing silica gel to minimise fungal growth in humid conditions. Samples were then shipped to the laboratory, where they were stored in a −20 °C freezer. GPS co-ordinates and other associated information (track marks, substrata etc.) were collected for each sample. Entire sampling was conducted once per site between January 2015 and June 2017 ensuring maximum coverage of the study area, covering PTR (257.3 km^2^), MTR (1,500.49 km^2^), NNTR (152.8 km^2^), TATR (627.5 km^2^) and UKWLS (189 km^2^), Maharashtra. A total of 249 samples were collected (PTR—92, MTR—76, NNTR—37, UKWLS—34, TATR—10, respectively) for this study. Details of all sample locations are given in Fig. 1.

For blood sampling, four animals (three males and one female) were remotely administered with reconstituted lyophilised mixture of Telatamine-Zolezepam (Zoletil 100; Virbac, Carros, France) at the dose rate of 8.6 mg/kg body weight using Dan-inject syringe projector (Model-IM) at a distance of 15–25 m. After ensuring sedation safe for handling, animal was approached, blindfolded and one ml of blood was collected through femoral-saphenous vein puncture. Blood was collected in EDTA vacutainers and preserved at −20 °C in the laboratory for genetic work.

### DNA extraction

DNA extraction was performed in the laboratory from the frozen faecal samples using QIAamp DNA Tissue Kit (QIAGEN Inc., Hilden, Germany) with a modified approach, depending on sample quality. If the sample had the entire top mucous layer available (i.e. not covered by dust, soil etc.) then it was swabbed with phosphate buffer saline soaked sterile cotton swab and was stored in sterile Eppendorf tube at −20 °C (Biswas et al., in press). However, if the mucous layer was covered then the top layer was scraped using sterile blades and stored in similar conditions (Biswas et al., in press). Subsequently, faecal samples collected by both methods were lysed overnight in 300/600 μl of lysis buffer for swabs and scraped samples, respectively and 20 μl proteinase K followed by extraction using the kit’s protocol. DNA was eluted twice with 100 μl of 1X TE and stored in −20 °C for long-term use. Each set of 22 extractions was accompanied with two negative controls to monitor possible contamination.

DNA from blood samples was extracted using standard protocol given in the QIAamp DNA Tissue Kit (QIAGEN Inc., Hilden, Germany). Negative control was incorporated to monitor any possible contaminations.

### Selection of microsatellite markers

There are no dhole specific microsatellites developed so far and the only study focusing on dhole population genetics had used 13 cross-species markers from domestic dogs to study genetic variation (Iyengar et al., 2005). These markers showed low levels of polymorphism and low PID_sibs_ value (3.3 × 10^−4^), providing a misidentification rate of 1 in 3,000 siblings. Given that India is considered to retain high (about 1,500–3,000) number of dholes (Kamler et al., 2015), this panel will not provide sufficient statistical power for unambiguous individual identification at landscape levels with large population sizes. For this study, we developed a panel following stringent cross-species marker selection and testing process. The entire process was conducted in two steps: marker selection and rigorous testing before developing a final microsatellite panel for dhole individual identification.

As most of the cross-species markers were found to be from dogs and earlier used markers were less polymorphic for individual identification, we decided to first examine if both species (domestic dog and wild dog) share genetic similarity. Earlier karyotype and chromosomal banding studies (Graphodatsky et al., 2008) showed almost identical G-banding patterns, indicating high chromosomal level similarity between both species. Subsequently, we identified a total of 37 dog microsatellite loci from earlier published literature (Holmes et al., 1995; Ostrander, Sprague & Rine, 1993; Fredholm & Winterø, 1995; Ostrander et al., 1995; Francisco et al., 1996; Neff et al., 1999). These markers were selected based on their polymorphism (number of alleles (Na), PIC, observed heterozygosity (H_o_) etc.) and amplicon sizes in published literatures. Further, we mapped all the markers on available dog genome canFam 3.1 in UCSC Genome Browser (http://genome.ucsc.edu/; Accession ID: GCA_000002285.2) to assess the chromosome number to which each marker is associated with. Finally, a total of 18 microsatellites were selected based on their amplicon size, chromosome number and polymorphism (based on published data) for further testing. The details of the markers are given in Table 1.

**Table 1:** Details of microsatellite loci used for Asiatic wild dog.

Locus	Primer sequence 5′–3′	Repeat motif	Dye	*T*_a_ (°C)	Chromosome number (in dog)	Na	H_o_	H_e_	Allelic range	PID_(unbiased)_ (cumulative)	PID_(sibs)_ (cumulative)	Success rate (%)	Genotyping error (%)			Reference
													ADO	FA	NA	
WD2201 (Panel 4)	ATCAACAATGCATGCCACAT GAGAACAAATAAATGCAAGCCC	Tetra	FAM	59	7	9	0.63	0.78	170–202	7.40*E*-02	3.81*E*-01	73.97	10.4	6.9	3.5	Francisco et al. (1996)
PEZ6 (Panel 3)	ATGAGCACTGGGTGTTATAC ACACAATTGCATTGTCAAAC	Tetra	NED	53	27	7	0.38	0.78	206–230	5.88*E*-03	1.46*E*-01	72.05	9.1	8.2	19.2	Neff et al. (1999)
WD2140^#^ (Panel 2)	GGGGAAGCCATTTTTAAAGC TGACCCTCTGGCATCTAGGA	Tetra	HEX	56	5	9	0.4	0.76	122–178	5.00*E*-04	5.69*E*-02	69.65	10.2	7.9	4.8	Francisco et al. (1996)
AHT130^#^ (Panel 2)	CCTCTCCTGGTAAGTGCTGC TGGAACACTGGTCCCCAG	Di	FAM	56	18	7	0.44	0.74	98–112	4.69*E*-05	2.29*E*-02	71.47	13.9	2.4	0	Holmes et al. (1995)
PEZ3 (Panel 3)	CACTTCTCATACCCAGACTC CAATATGTCAACTATACTTC	Tetra	PET	53	19	8	0.52	0.73	110–146	5.26*E*-06	9.53*E*-03	88.45	11	16.5	16.4	Neff et al. (1999)
WD2137 (Panel 4)	GCAGTCCCTTATTCCAACATG CCCCAAGTTTTGCATCTGTT	Tetra	FAM	56	3	7	0.46	0.66	156–180	7.29*E*-07	4.33*E*-03	68.94	9	1.6	22.1	Francisco et al. (1996)
PEZ5 (Panel 3)	GCTATCTTGTTTCCCACAGC TCACTGTATACAACATTGTC	Tetra	FAM	53	12	8	0.15	0.61	150–254	1.39*E*-07	2.14*E*-03	57.37	12.9	6.9	16	Neff et al. (1999)
CXX251 (Panel 2)	TACCACTGTCATTTTTCCATGC AAGAGGATACCGGTGGCAG	Di	NED	56	1	3	0.23	0.58	128–136	3.32*E*-08	1.11*E*-03	78.99	8.8	5.6	11	Ostrander et al. (1995)
WD2096^#^ (Panel 1)	CCGTCTAAGAGCCTCCCAG GACAAGGTTTCCTGGTTCCA	Tetra	FAM	59	11	3	0.66	0.51	93–101	1.05*E*-08	6.38*E*-04	81.98	8.6	8.5	24	Francisco et al. (1996)
CXX279^#^ (Panel 1)	TGCTCAATGAAATAAGCCAGG GGCGACCTTCATTCTCTGAC	Di	PET	59	22	5	0.2	0.45	125–135	3.29*E*-09	2.33*E*-04	84.05	7.0	6.9	13	Ostrander et al. (1995)
WD2001^#^ (Panel 1)	TCCTCCTCTTCTTTCCATTG TGAACAGAGTTAAGGATAGACAC	Tetra	HEX	59	23	3	0.41	0.48	134–142	1.24*E*-09	3.86*E*-04	78.92	10.4	8.2	6.2	Francisco et al. (1996)
CXX30 (Panel 1)	GCCTTTTAGGGAGCTTTCTTT GAGTCTGCTTTTCTCCTTCCC	Di	NED	59	2	3	0.38	0.41	122–142	4.68*E*-10	1.50*E*-04	77.28	9.4	9.9	3.3	Ostrander et al. (1995)
Mean						6	0.40	0.62				75.26	10.1	9.2	11.2	
AHT136*	GAGAGGGCTGGTGGTAGGGG CGTGGCTATCTTTGGAGGGA	Di	HEX	NA	11	–	–	–	–	–	–	–	–			Neff et al. (1999), Holmes et al. (1995)
WD2159*	GAATCCCACATCGGGCTC ATTAAGTTTTGAAAGCCAGGTAAG	Tetra	HEX	NA	24	–	–	–	–	–	–	–	–			Francisco et al. (1996)
CPH6*	CATTGGCTGTTTGACTCTAGG ACTGATGTGGGTGTCTCTGC	Di	FAM	56	23	4	0.189	0.577	107–136	–	–	–	–			Fredholm & Winterø (1995)
CPH16*	CTACACCAGTTAGGGAATCTAGC CAGATTCAAATCCACTCTCAGAC	Di	HEX	NA	20	–	–	–	–	–	–	–	–			Fredholm & Winterø (1995)
CXX140*	CAGAGGTGGCATAGGGTGAT TCGAAGCCCAGAGAATGACT	Di	PET	56	4	2	0.012	0.059	149–151	7.42*E*-08	9.87*E*-04	–	–			Ostrander et al. (1995)
CXX608*^#^	TATTGTAAGTCTTCCTTGAC TCTACCGTCTACAACAAAAGGG	Di	HEX	53	15	2	0	0.04	134	7.42*E*-08	9.87*E*-04	–	–			Ostrander et al. (1995)

**Notes:**

Na, number of alleles; H_o_, observed heterozygosity; H_e_, expected heterozygosity; PID, probability of identity; *T*_a_, annealing temperature; ADO, alleleic dropout; FA, false alleles; NA, null alleles.

* Loci omitted from final analyses.

# Loci used in the study by Iyengar et al. (2005).

### PCR standardisation and data validation

All initial standardisation of the markers was conducted using dhole blood samples (*n* = 4). PCR reactions were performed for selected 18 microsatellites in 10 μl reactions containing 3.5 μl Qiagen multiplex PCR buffer mix (QIAGEN Inc., Hilden, Germany), 0.2 μM labelled forward primer (Applied Biosystems, Foster City, CA, USA), 0.2 μM unlabelled reverse primer, four μM BSA and two μl of 1:50 dilution of blood DNA extract. The PCR conditions included an initial denaturation (95 °C for 15 min); 50 cycles of denaturation (94 °C for 30 s), annealing (50–60 °C gradient for 30 s) and extension (72 °C for 35 s); followed by a final extension (72 °C for 30 min). Following post-temperature standardisations markers with same annealing temperatures but with different labels or allele sizes were standardised as multiplex assays (see Table 1 for details). During all amplifications, both extraction and PCR negative controls (one PCR negative every set of 11 reactions) were included to monitor any possible contamination. Post amplification, two μl of PCR product was mixed with HiDi formamide (Applied Biosystems, Foster City, CA, USA) and LIZ 500 size standard (Applied Biosystems, Foster City, CA, USA) and genotyped in an ABI genetic analyser (Applied Biosystems, Foster City, CA, USA). The fragment lengths were scored manually using the programme GENEMARKER (Softgenetics Inc., State College, PA, USA). Each reaction was repeated three times to ensure good data quality.

Once the initial temperature and multiplexing standardisations were performed using reference blood DNA samples, final standardisation was conducted with dhole faecal DNA. Species identification was performed for all field-collected faeces using specific mtDNA primers described in Modi et al. (2018). PCR reactions were performed with four μl of hotstart taq mix (QIAGEN Inc., Hilden, Germany), four μM BSA, 0.5 μM of primer mix and three μl of DNA extract with conditions including initial denaturation (95 °C for 15 min); 50 cycles of denaturation (94 °C for 30 s), annealing (50 °C for 30 s) and extension (72 °C for 35 s); followed by a final extension (72 °C for 10 min). Negative controls were included to monitor contaminations. Samples that produced species-specific bands (*n* = 225) were further processed for microsatellite analyses.

For faecal samples, data validation was performed through a modified multiple-tube approach as described in Mondol et al. (2009b). All faeces that had amplified in 50% of the loci in the panel during first PCR were repeated two more times for all loci. Following allele calling, a consensus genotype was prepared using the ‘Quality index’ protocol (Miquel et al., 2006), during which alleles were called manually and scored as ‘1’ if the repeat is identical with the first call, or ‘0’ if calls do not match due to no amplification, allelic dropout (one allele in heterozygote is erroneously not amplified), false allele (FA; slippage artefact during PCR) etc. To calculate the quality index for each locus/sample the scores assigned to each repeat are summed and divided by the total number of repeats, and only quality index of 0.75 or more (at least three out of four repeats) for each locus was considered for downstream analyses. We calculated average amplification success as the percent positive PCR for each locus, as described by Broquet & Petit (2004). We quantified allelic dropout and FA rates manually as the number of dropouts or FAs over the total number of amplifications, respectively (Broquet & Petit, 2004), as well as using MICROCHECKER v 2.2.3. (Van Oosterhout et al., 2004). The FA frequency was calculated for both homozygous and heterozygous genotypes as the ratio of the number of amplifications having one or more FAs at a particular locus and the total number of amplifications while allele dropout rate was calculated as the ratio between the observed number of amplifications having loss of one allele and the number of positive amplifications of the heterozygous individuals. Programme FreeNA (Chapuis & Estoup, 2007) was used to determine the frequency of null alleles (NAs), which estimates the NA frequency using EM algorithm (Dempster, Laird & Rubin, 1977).

Molecular sexing of the identified individuals was conducted using already developed multiplex sexing approach (Modi et al., 2018), where three sex chromosome specific markers (DBY, AHT-X40 and SRY) were combined to generate a three-band pattern for males and a single band for females. This approach reduces identification of ‘false negatives’ of males due to allelic dropout from the Y chromosome from poor quality samples.

### Data analyses

The identity analysis module implemented in programme CERVUS (Kalinowski, Taper & Marshall, 2007) was used to identify identical genotypes (or recaptures) by comparing data from all samples for all amplified loci. All genetic recaptures were removed from the data set. GIMLET (Valière, 2002) was used to calculate the PID_(sibs)_ for all the individuals. Following this, any allele having less than 10% frequency across all amplified loci were rechecked for allele confirmation. ARLEQUIN (Excoffier, Laval & Schneider, 2005) was used to determine Hardy Weinberg equilibrium and linkage disequilibrium for all the loci.

## Results

During initial standardisations we tested all 18 selected markers (see Table 1) with four wild-caught dhole blood DNA samples. Three of these tested markers (WD2159, CPH16, AHT136) did not show any amplification in the blood DNA samples and were removed from subsequent analyses. The remaining 15 markers were then amplified with 225 genetically confirmed dhole faecal samples. Following data validation through multiple repeats, amplification success rates and polymorphism for these loci were calculated. The results show that loci CXX608 and CXX140 were monomorphic in all amplified samples, and locus CPH6 has low amplification success rate (~35%) from faecal DNA and thus were removed from the panel. The remaining 12 markers were finally standardised as four multiplex panels (see Table 1) for dhole individual identification.

None of these final 12 loci showed any signatures of large-scale allelic dropouts. The mean allelic dropout rate was found to be 0.1, whereas mean FA frequency for all the 12 loci was 0.092. Overall frequency of NAs was calculated as 0.11, indicating this 12 loci panel has low genotyping error rates. Amplification success ranged between 57% and 88% from dhole faecal DNA. The loci showed relatively higher (WD2201-9 alleles, H_o_ = 0.63) to medium (CXX251-3 alleles, H_o_ = 0.23) levels of polymorphism (Table 1). Except locus WD2001, none of the other loci were found to deviate from the Hardy–Weinberg equilibrium and there were no evidences for strong linkage disequilibrium between any pair of loci. Summary statistics for various measures of polymorphism (H_o_ and expected heterozygosity, Na and allelic size range) for all loci in the final panel are presented in Table 1.

**Figure 2:**
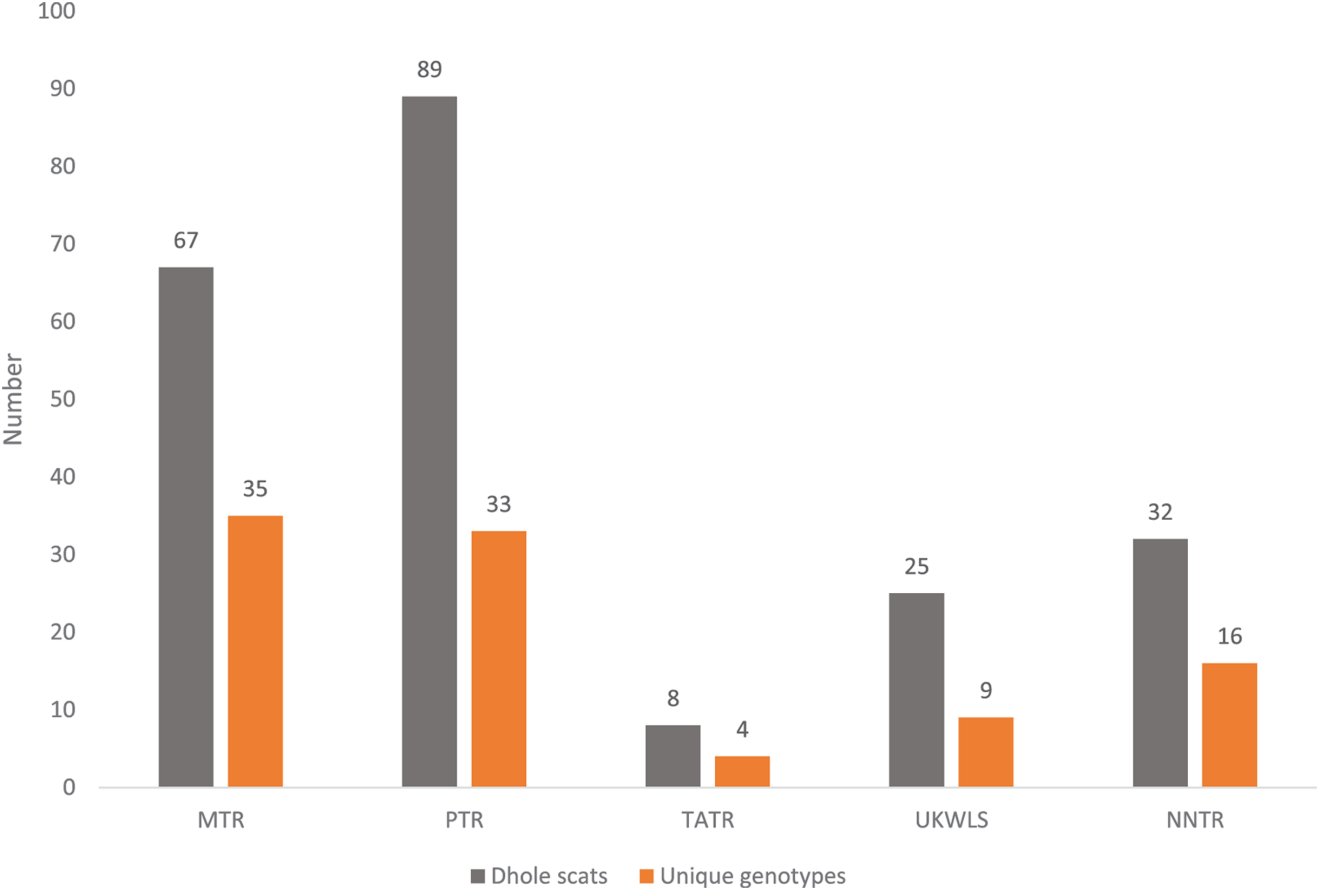
Graph showing the number of confirmed dhole scats collected from the field and area-wise unique genotypes identified from them.

For individual identification, we only considered samples that produced good quality data for at least seven of the 12 panel loci. This cut-off value of average of seven loci was decided based on the statistical support (PID_sibs_ value of 1 in 500 siblings) produced by these loci. Given that any single largest dhole population is about 250–300 individuals (Kamler et al., 2015), this value is sufficient for individual identification at local scales in India. Out of the 225 field-collected dhole faecal samples amplified with the panel of 12 markers, 98 produced seven or more loci data. Overall, we generated genetic data from a total of 102 samples (four blood and 98 faecal samples) (Fig. 1). About 70% of these samples (*n* = 71) have successfully amplified for 10–12 loci. Following analyses with CERVUS, we identified 101 unique dhole individuals from the entire data, whereas one individual from NNTR was found to be a ‘genetic recapture’. Cumulative PID_sibs_ and PID_unbiased_ values were found to be 1.5 × 10^−4^ and 4.7 × 10^−10^, respectively, indicating a strong statistical support for unambiguous individual identification. The number of unique individuals from each sampled area was found to be: PTR—33, MTR—35, NNTR—16, UKLWS—9 and TATR—4 (Fig. 2). Molecular sexing showed a success rate of 67%, with a male:female sex ratio of 4:1 in all identified dhole individuals (*n* = 101).

## Discussion

In this paper, we standardised protocols for individual identification of Asiatic wild dogs from poor quality DNA samples, and the final marker panel could unambiguously identify individual dholes in our field-based pilot study from five protected areas of Maharashtra, India. The systematic protocols followed here offer some advantages over earlier efforts on dhole individual identification from faecal samples by Iyengar et al. (2005). Firstly, use of a large panel of 37 microsatellite loci for preliminary assessment of marker suitability along with genomic mapping-based selection of final markers (*n* = 18) allowed us to ascertain a combination of loci for unambiguous individual identification with high statistical power. The rigorous testing of the loci with large number of DNA samples from different sources also allowed us to exclude loci that might be problematic due to low amplification success from non-invasive samples. The final panel consisting 12 markers were further standardised into four multiplex reactions to provide time and cost-effective options during data generation. We were very careful to initially select a large number of tetranucleotide markers as they are known to have low stutter peak problems and better allele characteristics from poor quality samples (Walsh, Fildes & Reynolds, 1996), while dinucleotide markers generally have higher amplification success (Broquet, Ménard & Petit, 2007). Thus, our final panel with a ratio of 2:1 tetra vs. dinucleotide microsatellites would provide the ideal combination in terms of high success rate and less technical issues in allele calling during dhole individual identification. The amplification success rate for all loci was >70% except locus PEZ5 (~60%), but it was found to be polymorphic and was included in the panel. The overall genotyping error frequency was found to be <0.2 from dhole faeces, which is within the recommended limits for non-invasive population genetic research (Smith & Wang, 2014).

Our motivation in this study was to develop effective protocols that could be applied for individual identification of Asiatic wild dogs as they are difficult to identify from physical characteristics (spots, marks, stripes etc.). Their elusive nature also makes it challenging to estimate population size using traditional techniques (photographic capture, field-based observations etc.) at landscape levels. For genetic estimation of population size Waits, Luikart & Taberlet (2001) recommended a threshold PID_sibs_ value that is at least double than the approximate number of animals in any given area. The cumulative PID_sibs_ value of 1.5 × 10^−4^ achieved in this study is better than Iyengar et al. (2005) (PID_sibs_ of 3.3 × 10^−4^) and should be sufficient to study dhole genetics and specifically population estimation across its range. Among all the dhole range countries India is considered to retain the highest (about 1,500–3,000) number of individuals (Kamler et al., 2015) and our misidentification rate achieved in this study (1 in 6,700 siblings) would provide strong statistical power in individual identification. The most recent assessment suggests that the largest dhole population in Western Ghats, India holds about 207–304 individuals (Kamler et al., 2015), thereby assuring that our seven loci cut-off (misidentification rate 1 in 500 siblings) to select samples and 12 loci panel would be useful in population estimation at local scale. However, it is important to point out that we generated individual level information from about 43.5% (98 out of 225 faeces) of the field-collected samples in this study. Similar patterns of low amplification success rate from field-collected faecal samples have been observed in earlier genetic study of dhole (Iyengar et al., 2005), leopard (Mondol et al., 2009a) and other species (Smith & Wang, 2014). Considering dhole cryptic nature, social behaviour and ecology in corroboration with low amplification success rate, we suggest an intensive faecal sampling effort for estimation of population size for this species. It is also noteworthy to point out that literature survey for dhole marker selection in this study was mostly based on available information on non-invasive canid population genetic research with specific information available on markers such as marker polymorphism (Na, H_o_) and amplicon size etc. (Holmes et al., 1995; Ostrander, Sprague & Rine, 1993; Fredholm & Winterø, 1995; Ostrander et al., 1995; Francisco et al., 1996; Neff et al., 1999). However, future studies should also consider additional markers those are tested as part canid forensic studies (for example see Van Asch et al., 2009; Berger et al., 2014; Hellmann et al., 2006; Eichmann, Berger & Parson, 2004) on dholes. In addition, already available dhole genome information (Campana et al., 2016; Habib et al., 2018) also can be used to develop a suitable SNP panel for in depth analyses of dhole population and demographic parameters.

During individual identification we had identified only one genetic recapture from the field-collected faecal samples. This pattern of low dhole recapture could be attributed to our sampling strategy to cover large geographical area and maximise collection of faeces from potentially different individuals, as well as relatively low amplification success rates from faecal samples. We have sampled the entire study area only once and focused on collecting fresh samples, thereby probably missed recapturing the same individuals from same latrine sites. Further, low amplification success rate (101 genotypes from 225 fresh samples) might have resulted in getting lesser number of recaptures. We got a male biased sex-ratio (4:1) in this pilot study. While there is no conclusive information on dhole sex ratio across its range, our earlier study (Modi et al., 2018) in the same landscape has shown a male biased (M:F ratio of 3:1) sex ratio, and ecological study in southern India by Venkataraman (1998) suggested male biased packs. Future studies with extensive genetic sampling across this landscape would potentially provide more accurate sex ratio for dholes.

## Conclusion

In the broader context of understanding dhole population dynamics at local or landscape scales, genetic sampling is possibly the only way to generate information with spatial and temporal coverage for this elusive, social carnivore as photographic sampling or conventional tagging cannot be employed due to lack of distinguishing natural marks and logistical difficulties of physical captures of large number of animals. Results from this study provide a robust tool to generate individual level information from field-collected faecal samples. In combination with a good sampling strategy, our methods can be used in a cost-effective way to investigate species biology (including patterns of genetic diversity, relatedness and population connectivity) as well as to estimate population abundance of dholes in the wild.

## Supplemental Information

10.7717/peerj.7453/supp-1 Supplemental Information 1 Raw genotype data for 101 dholes.

## Additional Information and Declarations

### Field Study Permissions

The following information was supplied relating to field study approvals (i.e. approving body and any reference numbers): Reference dhole blood samples (*n* = 4) were collected as part of another ongoing study in Tadoba-Andhari Tiger Reserve under Permit no. SPP-12/2016, granted by the Office of the Principal Chief Conservator of Forests (HOFF), Maharashtra State. Feacal sampling was done under Permit No. 09/2016, granted by the Principal Chief Conservator of Forests (Wildlife) & Chief Wildlife Warden, Maharashtra State.

### Data Availability

The following information was supplied regarding data availability: The raw data of microsatellite genotypes are available in the Supplemental File and Dryad: DOI 10.5061/dryad.17r4585.
